# Vitamin D Deficiency in Patients With Low-Energy Hip Fractures in Accordance With the Mediterranean Paradox

**DOI:** 10.7759/cureus.57583

**Published:** 2024-04-04

**Authors:** Christos Konstantinidis, Ourania Psoma, Christos Kotsias, Vasileios Panagiotopoulos, Sotiris Plakoutsis, Dimitrios Tsiampas, Dimitrios Vardakas, Dimitrios Giotis

**Affiliations:** 1 Orthopaedic Department, General Hospital of Ioannina "G. Hatzikosta", Ioannina, GRC; 2 Department of Internal Medicine, School of Medicine, University of Ioannina, Ioannina, GRC

**Keywords:** mediterranean paradox, hip fractures, low-energy trauma, deficiency, vitamin d

## Abstract

Introduction

Vitamin D deficiency (VDD) is considered one of the leading causes of poor bone quality. It may also be related to severe muscular weakness, especially in the elderly, which leads to frequent falls. Thus, VDD might be associated with fragility fractures of the hip, wrist, and spine in this age category. In this cross-sectional study, our goal was to present vitamin D levels in an elderly Mediterranean population with hip fractures and to assess whether its levels are related to the incidence or prevention of such injuries.

Methods

Between January and December 2021, 140 patients aged 65 years or older were hospitalized in our department with a fracture involving the hip joint. Serum calcium and vitamin D level control was performed upon admission, as well as recording whether anti-osteoporosis medication had been prescribed. Only patients with low-energy fractures were included, whereas oncologic patients and those with high-energy trauma were excluded.

Results

Thirty-eight men and 102 women, with a mean age of 83.12 and 84.88 years, respectively, participated in our study. Intertrochanteric fractures were the most common injuries (50.72%). Low vitamin D levels (<30 ng/mL) were observed in 132 patients (94.28%). A bone density scan during the last year had been conducted by only seven patients (5%), whereas in 136 patients (97.14%), no anti-osteoporotic medication was given.

Conclusion

There is an excessive percentage of aged patients with hip fractures in Greece, demonstrating a significant vitamin D insufficiency despite the high annual frequency of sunny days in this Mediterranean region. Presumably, most of these patients neither perform the routine bone density scan nor do they take any kind of preventive pharmaceutical treatment, which might reveal devaluation of osteoporosis from this age group due to contingent comorbidities.

## Introduction

Vitamin D is a fat-soluble secosteroid pleiotropic hormone found naturally in certain ailments. In particular, vitamin D3 (cholecalciferol) is highly provided by sea fish fat and cod liver oil, while D2 (ergocalciferol) is mainly contained in mushrooms and plants [1]. However, vitamin D3 is primarily synthesized in the skin from 7-dehydrocholesterol after exposure to daylight, especially to ultraviolet B radiation [1]. It is metabolized to 1,25(OH)2D3, the hormonally active form after two main hydroxylations. In the first step, cholecalciferol is hydroxylated to 25-hydroxycholecalciferol [25(OH)D3] within the liver and subsequently to the final active hormone 1,25(OH)2D3 within the kidneys. The cardinal role of 1,25(OH)2D3 is to control calcium and phosphorus balance in plasma through receptors in kidneys, intestines, and bones, regulating mineral and bone homeostasis [2].

Poor vitamin D levels are considered to trigger the equilibrium between osteogenesis and bone remodeling, resulting in rickets in children and osteopenia or osteoporosis in elderly patients [3]. Moreover, it has been observed that vitamin D deficiency (VDD) might also be related to severe muscle weakness, which may predispose to a higher risk of falls and, consequently, to an increased prevalence of fractures [4-6].

Over the last years, the term “osteoporotic fracture” has been replaced by terms like “fragility fracture” or “low-energy fracture,” indicating that without the underlying bone disease, minimal trauma would not normally result in such a type of injury [7]. Hypovitaminosis D (classified as VDD or vitamin D insufficiency) seems to be an independent risk factor for low-energy fractures [8]. Vitamin D inadequacy has been related to distal radius fractures in the elderly of both sexes regardless of bone mineral density (BMD), body mass index (BMI), or smoking history [9]. It has also been reported that patients (both genders) who suffer from vertebral fragility fractures are more likely to present VDD [10]. On the other hand, it has been noted that patients with VDD are more prone to hip fragility fractures [11,12], and their functional recovery and improvement of life quality within the first six postoperative months after a hip fracture is inferior in those with poorer vitamin D status compared to patients with normal vitamin D levels [13].

The purpose of the current study was to present vitamin D levels in an elderly Mediterranean population with hip fractures to assess whether this vitamin D inadequacy in this sunny region, known as the “Mediterranean Paradox,” could be related to the incidence of such injuries and possibly if adequate levels of this vitamin could assist in their prevention. A review of literature regarding this phenomenon was also conducted. Thus, it was hypothesized that patients with low-energy hip fractures would present low vitamin D concentrations despite living in a region with adequate sunlight exposure.

## Materials and methods

From January to December 2021, patients with hip fragility fractures who were admitted to our General Regional Hospital participated in this cross-sectional prospective study. One hundred forty patients were included in the study who had sustained an intertrochanteric, subtrochanteric, or femoral neck fracture after low-energy trauma, namely a fall from a standing height or lower. The diagnosis of the hip fracture was performed with radiographs or even CT scans in controversial cases. On the contrary, patients with pathologic or periprosthetic fractures, metabolic bone diseases other than osteoporosis, and fractures caused by a high-energy mechanism of injury were excluded from the study.

Venous blood samples were acquired from all patients for biochemical analysis in a fasting state at least eight hours after their last meal. These were used for measuring serum calcium and 25(OH)D3. The latter, being the active form of vitamin D, is considered the main indicator of vitamin D status [2]. The reference range of serum calcium, as determined by our biochemistry lab, was 8.2-10.5 mg/dL. Moreover, in accordance with the 2011 guidelines of the Endocrine Society, VDD is defined as a serum 25-hydroxy-vitamin D concentration of less than 20 ng/mL, a level of 21-29 ng/mL as insufficiency and values above 30 ng/mL are regarded as sufficiency [14]. In addition, all patients obtained a thorough medical history, with emphasis given on previous anti-osteoporotic medication and vitamin D supplements.

All procedures conducted in the study were in agreement with the Helsinki Declaration of Principles of 1964, as revised in 2008 [15]. The study was reviewed and approved by the institutional review board of the hospital, and all patients agreed to participate by signing informed consent forms. Statistical analysis of all data collected was performed using the IBM SPSS Statistics, version 26.0 (IBM Corp., Armonk, NY, USA), including a descriptive analysis of data that involved distribution, measures of central tendency, and measures of variability.

## Results

The mean age of the 140 patients included in the study was 84.61 ± 12.40 years (range, 65-99 years). In all, 27.14% (n = 38) were male, and 72.86% (n = 102) were female, with a male-to-female ratio of 1:2.69. Approximately half of hip fractures were intertrochanteric [50.72% (n = 71)], whereas the rest 42.14% (n = 59) and 7.14% (n = 10) were femoral neck and subtrochanteric fractures, respectively. The most common injury mechanism was a fall from a standing height while being at home [62.14% (n = 87)], whereas 25% of fractures (n = 35) had occurred in patients’ homes and outdoors and 12.86% (n = 18) in the street. The average length of hospital stay was 6.89 ± 3.85 days, specifically 6.82 ± 3.55 days for males and 6.92 ± 3.97 days for females (Table 1).

**Table 1:** Patients’ demographic data Mean and standard deviation values for age, length of hospital stay, average serum calcium, and average serum 25-OH-D3.

Parameters	Male (n = 38)	Female (n = 102)	Total (n = 140)
Age	83.12 ± 9.95	84.88 ± 14.76	84.58 ± 12.40
Intertrochanteric fractures, n	15 (39.47%)	56 (54.90%)	71 (50.72%)
Femoral neck fractures, n	22 (57.90%)	37 (36.28%)	59 (42.14%)
Subtrochanteric fractures, n	1 (2.63%)	9 (8.82%)	10 (7.14%)
Length of hospital stay in days	6.82 ± 3.55	6.92 ± 3.97	6.89 ± 3.85
Average serum calcium (mg/dL)	9.02 ± 0.74	8.96 ± 0.71	8.97 ± 0.72
Average serum 25-OH-D_3_ (ng/mL)	13.54 ± 7.82	13.98 ± 9.09	13.86 ± 8.74
Vitamin D deficiency, n (%)	30 (78.95%)	78 (76.47%)	108 (77.14%)
Vitamin D insufficiency, n (%)	6 (15.79%)	18 (17.65%)	24 (17.14%)
Vitamin D sufficiency, n (%)	2 (5.26%)	6 (5.88%)	8 (5.71%)
No anti-osteoporotic medication, n (%)	38 (100%)	98 (96.08%)	136 (97.14%)
Vitamin D supplementation, n (%)	1 (2.63%)	11 (10.78%)	12 (8.57%)

**Figure 1:**
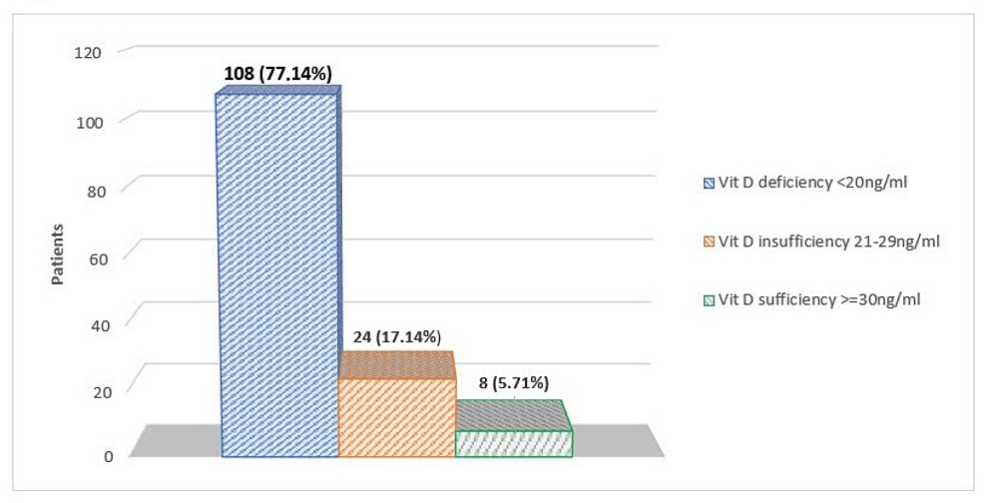
Patients’ categorization according to vitamin D (Vit D) concentrations

Regarding the level of 25-OH-D3, the majority of patients, in particular 77.14% (n = 108), had a serum 25-OH-D3 concentration lower than 20 ng/mL, thus rendering VDD predominant. Among the 108 patients with VDD, 30 (78.95%) were men and 78 (76.47%) were women. The average serum 25-OH-D3 concentration in men and women was 13.54 ng/mL (range, 3-37.1) and 13.98 ng/mL (range, 3-50.7), respectively. Vitamin D insufficiency was observed in 17.14% of patients (n = 24). Only eight patients (5.71%) had vitamin D sufficiency, demonstrating normal 25-OH-D3 levels (Figure 1).

Concerning serum calcium, the mean value was 8.97 ± 0.72 mg/dL, and a total of 14.28% of patients (n = 20) had values lower than the reference range (8.2-10.5 mg/dL). Furthermore, it was found that 19.28% of patients (n = 27) had a past history of low-energy fracture, and the most frequent preexisting medical comorbidities were hypertension (n = 103 [73.57%]), heart disease (n = 55 [39.29%]), diabetes mellitus (n = 41 [29.29%]), and depression (n = 39 [27.86%]) (Table 2).

**Table 2:** Patients’ comorbidity profile COPD, chronic obstructive pulmonary disease

Preexisting comorbidities	n (%)
Hypertension	103 (73.57)
Heart disease	55 (39.29)
Diabetes mellitus	41 (29.29)
Depression	39 (27.86)
Dyslipidemia	32 (22.86)
Hypothyroidism	24 (17.14)
Gouty arthritis	23 (16.42)
Cancer	10 (7.14)
Alzheimer disease	8 (5.71)
Parkinson disease	6 (4.29)
COPD	5 (3.57)

Remarkably, it was also recognized that the vast majority of patients, comprising a total of 97.14% (n = 136), had not received any anti-osteoporotic medication for at least one year prior to the hip fracture, while only 12 (8.57%) patients were receiving vitamin D supplements at the time of hospital admission. Moreover, it was observed that a bone density scan during the last year had been conducted by only 5% (n = 7).

## Discussion

The results of the present cross-sectional study demonstrated that elderly patients with a low-energy hip fracture who live in a sunny Mediterranean place display poor vitamin D levels in accordance with the “Mediterranean Paradox,” confirming our initial hypothesis. Interestingly, it was also found that very few patients were receiving vitamin D supplements, and even fewer were under medical treatment with anti-osteoporosis drugs. Furthermore, it was also observed that the percentage of patients who were evaluated for bone density over the last year was very low, as only in one out of 20 patients, bone mineral content (BMC) and BMD measurements were made by dual-energy X-ray absorptiometry (DEXA).

These vitamin D concentrations have been surprisingly low for a population living in an area cherished by the sun, with a mean of 266 sunny days per year. However, this phenomenon, referred to in the literature as the “Mediterranean Paradox,” has been widely observed, generating questions about potential root causes. Thus, Kyriakaki and Fragkoulis demonstrated poor vitamin D status in postmenopausal women living in Athens, one of the warmest and sunniest European capital cities [16]. An additional study carried out in Murcia, another Mediterranean city in south-eastern Spain known for its hot and sunny climate, presented similar findings emphasizing the prevalence of VDD and unexpectedly low levels of vitamin D, especially during summer months [17]. In parallel, Bettica et al. displayed an analogous high prevalence of hypovitaminosis D among postmenopausal women in Italy, another Mediterranean country [18]. However, vitamin D inadequacy has been noticed in other regions with abundant exposure to ultraviolet radiation, like in the Middle East and South Asia or in the southwest United States, where VDD has also been observed in patients with hip fractures [19,20].

The prevalence of VDD in our elderly population can be attributed to a plethora of different reasons. Basically, decreased exposure to sunlight is common for older patients who spend most of their time indoors due to coexisting comorbidities. In addition, in our study, it was shown that the vast majority of patients had not performed the routine bone density scan, and therefore, many of them were not taking any kind of preventive pharmaceutical medication, revealing devaluation of osteoporosis from this age group probably due to contingent comorbidities. Moreover, factors such as low dietary intake, obesity, industrialization of rural regions, and changes in lifestyle could also be accused of poor vitamin D status in many citizens [21,22]. In parallel, a strong association between polypharmacy and VDD has also been reported, especially among geriatric patients [23]. Furthermore, renal or liver conditions and malabsorption might also affect vitamin’s bioavailability in human skeletal and muscular cells, whereas anti-epileptic medications and corticosteroids can increase vitamin D catabolism. Lastly, primary hyperparathyroidism and hyperthyroidism have also been related to decreased levels of vitamin D [24].

On the contrary, according to the Velestino study that was conducted in Central Greece, VDD, even in sunny regions, might have a genetic background and might be associated with gene polymorphisms of vitamin D receptor (VDR) in a cumulative effect of three different genotypes, BsmI, TaqI, and Fokl. Specifically, patients who carried either B or T allele were more prone to VDD compared to the reference allele. Additionally, the accumulation of one, two, or all three of the above genotypes amplified the risk of developing VDD, underlining the missing piece of the puzzle for vitamin D inadequacy and explaining the paradox in regions with high sunlight exposure, like in our study [25].

This aforementioned VDD, particularly in the elderly population, can lead to low bone mass and subsequently to an increased risk of falls, predisposing to osteoporotic fractures [3-6]. Among others, hip fragility fractures are related to substantial morbidity and mortality in elderly patients, constituting a severe but occasionally fatal consequence of osteoporosis [26,27]. Jamal et al. reported that VDD seems to be a significant risk factor for low-energy hip fractures, as they noted a significant correlation between the degree of VDD and the severity of intertrochanteric fractures [28]. Chiang et al. demonstrated that the prevalence of VDD and insufficiency was strongly associated with hip fractures in older Taiwanese patients [29]. In general, high percentages of VDD in elderly patients with hip fractures have been detected in heterogeneous geographical regions varying from 50 to nearly 60% [30,31].

Aside from the increased incidence of hip fractures in patients with VDD, the preventive role of vitamin D in bone mineralization and, hence, in the prevention of low-energy fractures has been meticulously investigated [32-34]. It has been reported that VDD might lead to decreased mineralization of bone structure, causing osteomalacia in adult populations. Achieving equilibrium in serum vitamin D and parathyroid hormone (PTH) could prevent mineralization defects and secure the reduction of femoral fracture frequency [34]. A Cochrane review by Avenell et al. concluded that although vitamin D supplements could not generally prevent fractures, the combined administration of calcium and vitamin D seemed to reduce the risk of hip fractures in institutionalized elderly patients (RR, 0.84; 95% CI, 0.73 to 0.96) [32].

Vitamin D might also be related to improved neuromuscular function that could prevent instability in elderly individuals. The meta-analysis of Bischoff-Ferrari et al. concluded that there was a 22% reduction in falls as well as ameliorated walking and sit-to-stand test results in individuals receiving vitamin D supplements compared to placebo [33]. Falling events are a particularly important element in the etiology of hip fragility fractures [35]. Dretakis and Igoumenou found that patients with VDD display a significantly higher incidence of recurrent falls as compared to vitamin D non-deficient patients, with repeated fallers being correlated with trochanteric fractures and strengthened the belief that vitamin D inadequacy ends up in falls and potentially low-energy fractures [36].

Moreover, a meta-analysis by Kong et al. noted that daily administration of vitamin D dose of 800-1000 IU was related to decreased prevalence of falls and fractures [37]. The missing link between the incidence of falls and VDD could be sarcopenia, a geriatric syndrome characterized by the progressive loss of skeletal muscle mass and physical strength. The pathogenic background of sarcopenia involves a decrease in the number and size of muscle cells, especially type II, with a progressive infiltration of muscle fibers by connective and adipose tissue. VDD might be associated with these muscle abnormalities, while vitamin D supplementation could generate myogenesis and consequently increase the size and strength of type II fibers [38].

Regarding the economic aspects, hip fragility fractures place a substantial financial burden on the health and social care systems of countries faced with rapidly aging populations [39]. According to Neale et al., in Australia, 8% of hospitalizations for falls and hip fractures in older adults could be attributed to VDD [40]. In addition, Stevens and Lee demonstrated that mitigating VDD could prevent $247 million in medical costs annually among community-dwelling elderly [41]. Poole et al. claimed that the empirical treatment of middle-aged patients with vitamin D doses of 800 IU daily could lead to a significant cost-saving to the United Kingdom national health system of 1.2 £ billion over five years, as it could prevent about 620,000 minor and major falls, 84,000 person-years in long-term care, and a large number of deaths [42]. In parallel, Hiligsmann et al. observed that the treatment cost of vitamin D and calcium for adults of both sexes with osteoporosis was less than the cost of treating osteoporotic fractures in patients who were not receiving any supplementation [43]. Zarca et al. compared the cost-effectiveness of four different vitamin D supplementation strategies applied to a French elderly population who had never experienced a hip fracture before [44]. They noticed that for a treatment cost below 20 €, screening and treating VDD according to vitamin D levels was a highly cost-effective policy, highlighting the economic impact of primary prevention of hip fractures with vitamin D administration.

The effect of VDD on morbidity and postoperative quality of life in patients with a hip fracture has also been assessed. It has been reported that Vitamin D inadequacy can lead to high morbidity and disability after hip fracture surgery [27,45-47]. More specifically, Hao et al. revealed that patients with preoperative serum vitamin D levels below 12 ng/mL presented lower walking rates at 30 and 60 days after surgery than those with higher vitamin D concentrations [45]. Lim et al. similarly highlighted that preoperative VDD was not only related to the decrease in postoperative ambulatory status but also to the prolonged duration of hospitalization and increased risk of complications, such as delirium, pneumonia, and thromboembolism [46]. Likewise, LeBoff et al. found that decreased vitamin D levels at the time of the fracture were associated with poor lower extremity task performance and a high risk of falls one year after a hip fracture [27]. Guerra et al. underlined an even worse outcome of VDD, demonstrating that circulating levels below 12.5 ng/mL were correlated with an increased mortality rate of elderly patients after hip fracture surgical treatment within the first postoperative year [47]. 

Even if there is no global consensus regarding vitamin D supplementation dosage in VDD cases, current literature dictates that a lower daily dose may be superior to large monthly or annual doses in vitamin D bioavailability over time [48]. The Institute of Medicine (IOM) clarified that a daily dose of up to 4,000 IU showed no signs of toxicity [49]. In the current study, the vast majority of patients (87%) were treated with an oral dose of 2,000 IU for up to three months postoperatively, in order to reach and maintain the above 20 ng/mL threshold of serum vitamin D levels, whereas a second check of levels was proposed after the treatment.

To our knowledge, there is a scarcity in the literature regarding the correlation between the incidence of low-energy hip fractures in elderly patients and vitamin D levels in Mediterranean regions, which are paradoxically low despite the high sunlight exposure. Our study strongly associates this correlation. The general reluctance of these older persons to comply with medical guidelines for screening and supplementation is also highlighted. 

However, there are certain limitations in our study, such as the relatively small number of patients at a single center in a specific geographical area of Northwestern Greece and the lack of an in-hospital DEXA scan. Furthermore, the measurement of PTH levels could not be obtained for all patients since it was strictly performed once a week in our laboratory. There were also difficulties with the follow-up procedure since a considerable number of patients were residents of remote mountainous settlements. Nevertheless, a thorough medical history was obtained, and serum vitamin D and calcium levels were measured in all cases.

## Conclusions

VDD appears to be highly prevalent among patients with low-energy hip fractures. There is a strong correlation between VDD and osteoporosis, as well as muscular atrophy in elderly patients, which could lead to an increased incidence of falls, mainly in domestic environments. Even in geographical areas with adequate sunlight, elderly persons remain mostly homebound, thus not getting requisite sun exposure. Additionally, simple and potentially beneficial screening tests of vitamin D serum levels and DEXA are systematically neglected by patients or their relatives. Although there is no strong quality evidence of optimal vitamin D levels or proposed supplementation dosage, a yearly screening and evaluation of vitamin D should be conducted in order to enhance bone strength, muscular status, and overall healthcare.
